# Leprosy – eliminated and forgotten: a case report

**DOI:** 10.1186/s13256-019-2198-1

**Published:** 2019-09-01

**Authors:** Shiva Raj K.C., Geetika K.C., Purnima Gyawali, Manisha Singh, Milesh Jung Sijapati

**Affiliations:** 1 Department of Pathology, KIST Medical College, Imadol, Lalitpur, Nepal; 2 KIST Medical College, Imadol, Lalitpur, Nepal; 3 Department of Dermatology, KIST Medical College, Imadol, Lalitpur, Nepal; 4 Department of Internal Medicine, KIST Medical College, Imadol, Lalitpur, Nepal; 5 Department of Pathology and Laboratory Medicine, Patan Academy of Health Sciences, Lagankhel, Lalitpur, Nepal

**Keywords:** Erythema, *Mycobacterium*, Nodosum, Still

## Abstract

**Background:**

Leprosy is a disease that was declared eliminated in 2010 from Nepal; however, new cases are diagnosed every year. The difficulty arises when the presentation of the patient is unusual.

**Case presentation:**

In this case report we present a case of a 22-year-old Tamang man, from the Terai region of Nepal, with a clinical presentation of fever, malaise, and arthralgia for the past 2 weeks with hepatosplenomegaly and bilateral cervical, axillary, and inguinal lymphadenopathy. Features of chronic inflammation with elevated erythrocyte sedimentation rate of 90 mm/hour and liver enzymes were noted. With no specific investigative findings, a diagnosis of Still’s disease was made and he was given prednisolone. On tapering the medication, after 2 weeks, the lymphadenopathy and fever reappeared. On biopsy of a lymph node, diagnosis of possible tuberculosis was made. On that basis anti-tuberculosis treatment category I was started. During his hospital stay, our patient developed nodular skin rashes on his shoulder, back, and face. The biopsy of a skin lesion showed erythema nodosum leprosum and he was diagnosed as having lepromatous leprosy with erythema nodosum leprosum; he was treated with anti-leprosy medication.

**Conclusion:**

An unusual presentations of leprosy may delay its prompt diagnosis and treatment; thus, increasing morbidity and mortality. Although leprosy has been declared eliminated, it should not be forgotten and physicians should have it in mind to make it a differential diagnosis whenever relevant.

## Introduction

Leprosy, also known as Hansen’s disease, is a chronic granulomatous disease caused by the bacteria *Mycobacterium leprae*. It primarily affects the peripheral nerves and skin. According to the World Health Organization (WHO), individuals having one of the following three features have leprosy: (i) definite loss of sensation in a pale (hypopigmented) or reddish skin patch; (ii) a thickened or enlarged peripheral nerve with loss of sensation; and (iii) the presence of acid-fast bacilli in a slit-skin smear [1]. Even with the declaration of the elimination of leprosy in 2010, more than 3000 new cases have been diagnosed, which makes it necessary for us to consider it a differential diagnosis [2]. Sometimes, the disease has an unusual presentation, crippling complications, and can be contagious, so its timely diagnosis and management are extremely important [3].

Here we present a case of a 22-year-old man with misleading primary presentation of fever, malaise, and arthralgia, and he had generalized lymphadenopathy along with hepatosplenomegaly. With the above-mentioned findings and the presence of leukocytosis along with neutrophilia, elevated C-reactive protein (CRP), and abnormal liver function test, he was diagnosed as having adult-onset Still’s disease (AOSD) and treated accordingly. However, during the course of management, he developed nodules over his shoulder, back, and face. A biopsy from the skin lesion finally revealed erythema nodosum leprosum (ENL).

## Case presentation

A 22-year-old Tamang man, from Terai region of Nepal, presented to the medical out-patient department (OPD) of KIST Medical College Teaching Hospital, Lalitpur, on 8 February 2017 with complaints of fever, malaise, and arthralgia for the past 2 weeks and dry cough for the past 5 days. The fever was associated with chills but no rigor, the maximum temperature was not recorded, and a non-itchy erythematous skin lesion on his left foot was seen 2 days prior to OPD visit. His past medical history was uneventful. He did not give any significant family history. There was no history of contact with patients with tuberculosis. There was no history of loss of appetite or weight loss; he was not on any medication. There was no history of the use of long-term medications.

On examination, he was febrile with a temperature of 37.8 °C (100 °F). A general physical examination revealed bilateral cervical, axillary, and inguinal lymphadenopathy and hepatosplenomegaly. Other systemic examination was unremarkable. No skin lesion was visible at the time of examination. Routine investigations, which included complete blood count (CBC) with erythrocyte sedimentation rate (ESR), liver function test, CRP, and antinuclear antibody (ANA) tests, were performed. He had leukocytosis (21,700/mm^3^) with neutrophilia (19,300/mm^3^) and raised ESR of 90 mm/hour. Serum glutamic oxaloacetic transaminase (SGOT) was elevated at 154 (normal, 12–78 IU/L) and serum glutamic pyruvic transaminase (SGPT) was elevated at 210 (normal, 46–116 IU/L). Total bilirubin and total protein were within normal limits. CRP was positive whereas the results of ANA and rheumatoid arthritis (RA) factor tests were negative. Other tests done were urine routine examination; the result of which was unremarkable. Urine culture, blood culture, and sputum culture were negative for pathogenic microorganisms. Since brucellosis and scrub typhus are endemic, serological tests were performed for *Brucella* antigen and scrub typhus along with hepatitis B virus and human immunodeficiency virus (HIV). All serological tests performed in our patient were negative. A serum adenosine deaminase (ADA) test was also performed to rule out tuberculosis and was within normal limits. A chest X-ray and computed tomography scan of his chest and abdomen were done and were unremarkable. During the first week of his hospital stay, he was treated with acetaminophen for fever. Since the fever did not subside and a definite diagnosis was not formulated with the above-mentioned tests, further investigations were ordered with a provisional diagnosis of pyrexia of unknown origin (PUO). Bone marrow aspiration and a biopsy were performed which showed myeloid hyperplasia suggesting inflammatory pathology. He was treated with broad-spectrum antibiotics and corticosteroids.

During his hospital stay, he developed skin rash at extensor surface of his foot. An incisional biopsy of the skin was performed and sent to histopathology. The skin biopsy was superficial and was not adequate for reporting. Hence, a repeat biopsy was recommended. However, our patient did not give permission to perform a re-biopsy. The timeline of our patient from the initial presentation is shown in Table 1. With present findings, that is, persistent fever, malaise, arthralgia, generalized lymphadenopathy, persistent leukocytosis with neutrophilia, elevated SGOT and SGPT, positive CRP, and negative ANA and RA factor, Still’s disease was suspected as it met almost all the criteria (Table 2).

**Table 1:** Timeline of the patient from the initial presentation

8 February 2017	The patient visited our hospital. Admitted to evaluate the cause of fever
14 February 2017	ENT consultation for cervical lymphadenopathy FNAC of cervical lymph node: reactive lymphadenitis
22 February 2017	The patient developed a skin lesion over his lower leg. Dermatological consultation was done. A skin biopsy was performed. The biopsy was inadequate and repeat was advised.
26 February 2017	A provisional diagnosis of pyrexia of unknown origin was made. Bone marrow aspiration with a biopsy was performed. Bone marrow showed myeloid hyperplasia suggesting inflammatory pathology.
2 March 2017	A diagnosis of adult-onset Still’s disease was made. Prednisolone 40 mg was started.
6 March 2017	Fever subsided and the patient was discharged
29 March 2017	Fever reappeared with cervical lymphadenopathy
29 March 2017	FNAC inguinal lymph node: granulomatous lymphadenitis
3 April 2017	ATT started
8 April 2017	Nodular skin rash on shoulder, back, and face developed
10 April 2017	Skin biopsy: erythema nodosum leprosum
18 April 2017	Referred to Anandaban Leprosy Hospital
18 April 2017	Slit-skin smear: 4+
18 April 2017	Diagnosed as lepromatous leprosy with erythema nodosum leprosum
18 April 2017	MBMDT with prednisolone

*ATT* anti-tuberculosis treatment, *ENT* ear, nose, and throat, *FNAC* fine-needle aspiration cytology, *MBMDT* multibacillary multidrug therapy cytology

**Table 2:** Diagnostic criteria for adult-onset Still’s disease [4, 5]

Yamaguchi’s criteria [4]	Fautrel’s criteria [5]
Major criteria	Major criteria
Fever > 39 °C, lasting 1 week or longer	Spiking fever ≥ 39 °C
Arthralgia or arthritis, lasting 2 weeks or longer	Arthralgia
Typical rash	Transient erythema
Leukocytosis > 10,000/mm^3^ with > 80% polymorphonuclear cells	Pharyngitis
	Polymorphonuclear cells ≥ 80%
	Glycosylated ferritin ≤ 20%
Minor criteria	Minor criteria
Sore throat	Maculopapular rash
Recent development of significant lymphadenopathy	Leukocytosis ≥ 10,000/mm^3^
Hepatomegaly or splenomegaly	
Abnormal liver function tests	
Negative tests for antinuclear antibody (IF) and rheumatoid factor (IgM)	
Exclusion criteria	Exclusion criteria
Infections	Infections
Malignancies (mainly malignant lymphoma)	Malignancies (mainly malignant lymphoma)
Other rheumatic disease (mainly systemic vasculitides)	Other rheumatic disease (mainly systemic vasculitides)
Five or more criteria are required, a minimum of two should be major criteria	Four or more criteria are required with minimum three major criteria

*IF* immunofluorescence

Our patient was prescribed 40 mg of orally administered prednisolone. With the administration of steroids, his fever subsided and his enlarged lymph node started to shrink. Because of a satisfactory response to a high dose of steroids, he was discharged. After 2 weeks of 40 mg of prednisolone, a tapering dose was initiated. Once he reached 20 mg dose of prednisolone, his fever and lymphadenopathy recurred. He revisited our OPD and with the possibility of collagen vascular disease was referred to a hematologist in another hospital.

The hematologist recommended performing a lymph node biopsy. His inguinal lymph node was excised and sent to histopathology. On microscopic examination, the lymph node had an intact capsule with partial effacement of the nodal architecture. Occasional histiocytic granulomas were seen in the lymph nodes. Caseous necrosis or Langhans type of multinucleated giant cells was not seen. Other areas showed lymphoid follicles with prominent germinal center. With the presence of granulomas and tuberculosis being quite common, the possibility of tuberculosis was considered and on that basis anti-tuberculosis treatment category I was initiated. The anti-tubercular treatment category I include 2 months of isoniazid, rifampicin, pyrazinamide, and ethambutol, followed by 4 months of isoniazid and ethambutol.

**Fig. 1:**
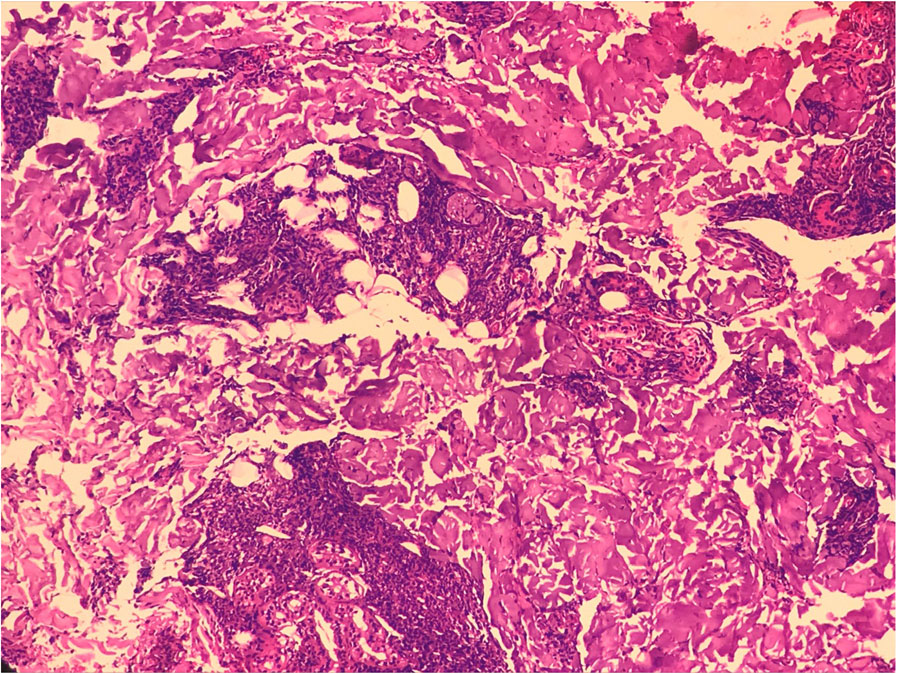
Photomicrograph showing patchy infiltration by epithelioid cells and lymphoid population along with foam cells and neutrophils. The infiltration was mainly limited to perineural and around the sweat glands and arrector pili muscles. (Hematoxylin stain; × 100)

**Fig. 2:**
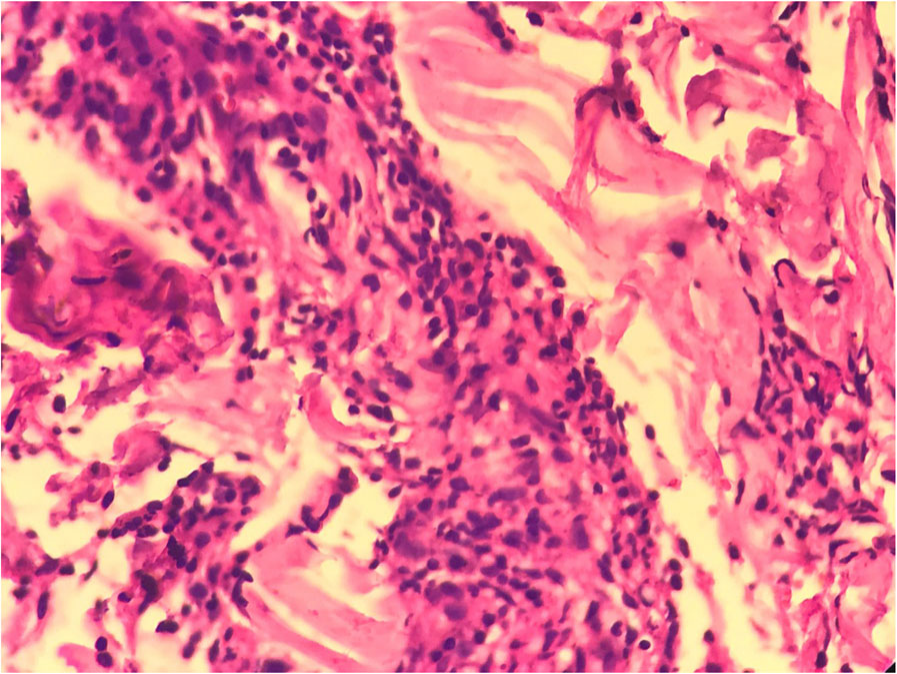
High-power view showing perineural infiltration by inflammatory cells. (Hematoxylin stain, × 400)

**Fig. 3:**
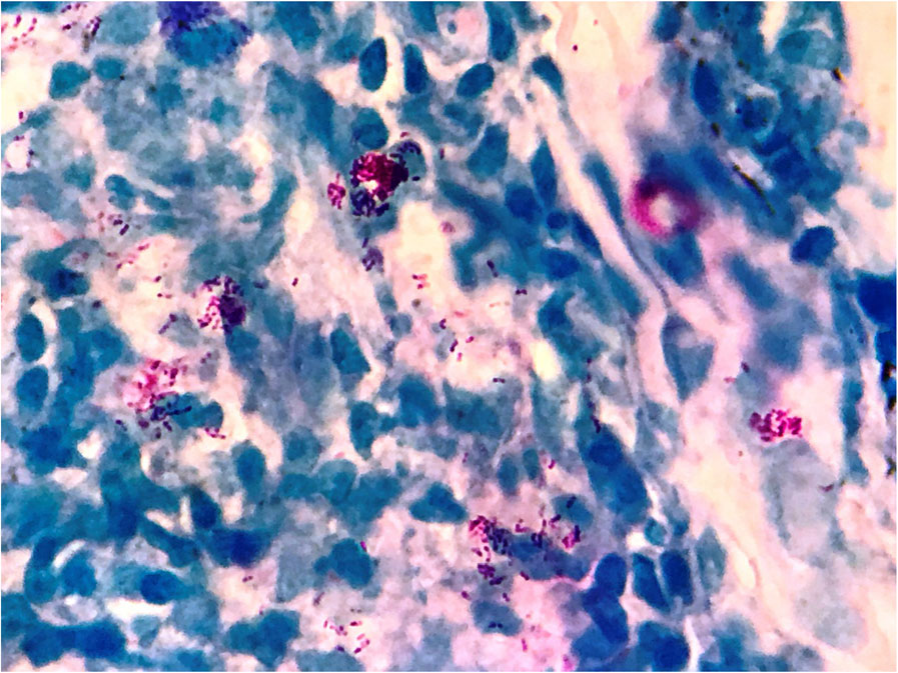
Photomicrograph showing several clusters of lepra bacilli (Wade Fite stain; × 1000)

During that period, our patient developed nodular rash at the shoulder, back, and face. A skin biopsy was performed on 10 April 2017, which showed superficial as well as deeper dermis with patchy infiltration by epithelioid cells and lymphoid populations, along with foam cells and neutrophils. The infiltration was mainly limited to perineural and around the sweat glands and arrector pili muscles. Subcutaneous tissue also showed infiltration by similar population of cells (Figs. 1 and 2). Wade-Fite stain in the tissue section showed several acid-fast bacilli (Fig. 3). He was diagnosed as having ENL. Anti-tubercular treatment was stopped and he was referred to Anandaban Leprosy Hospital, Lalitpur, Nepal.

In Anandaban Leprosy Hospital, a detailed history was taken retrospectively. There was no contact history and previous history of treatment for leprosy. He gave a history of transposition of ulnar nerve when he was 12 years of age, although he did not know the cause and no medical file was available. On examination, many erythematous lesions along with ENL nodules were present all over his body. There was a loss of eyebrows. Both left ulnar and left tibial nerve thickening was observed. There was mild clawing of his left little finger. A slit-skin smear showed a bacillary index of 4+. He was diagnosed as having lepromatous leprosy (LL) with ENL. He was admitted in the leprosy hospital and prescribed multibacillary multidrug therapy which constitutes standard regimen of rifampicin 600 mg once a month, dapsone 100 mg daily, and clofazimine 300 mg once a month and 50 mg daily. All these drugs are prescribed for 12 months. He is tolerating the prescribed medicine well and is showing improvement.

## Discussion

Leprosy, which was a major public health problem of Nepal, achieved its elimination in December 2009 and was declared eliminated in 2010 [2]. However, the incidence of new cases and prevalence rate has increased during fiscal years 2067/68 to 2073/74 to 0.79, 0.85, 0.84, 0.83, 0.89, 0.89, 0.92, respectively [6]. In Nepal, most of the patients with leprosy are referred to two leprosy hospitals. Because of the rare incidence of new cases of leprosy and in the presence of an unusual presentation, the differential diagnosis of leprosy may not be on the list. This may mislead the treating physician to search for other probable diagnoses.

Still’s disease is a systemic inflammatory disease of unknown etiology and pathogenesis; AOSD is quite rare and presents in 5 to 10% of patients with PUO. In 1897, George Still first described signs of symptoms of the disease in 22 children; hence, it was named Still’s disease. In 1971, Eric Bywaters described 14 adults with a presentation similar to that of pediatric Still’s disease, convincingly establishing a new disease entity [7]. AOSD affects more commonly younger individuals with almost three quarters of the reported cases of patients being between 16 and 35 years of age [8–10].

AOSD is characterized by the classic triad of persistent high spiking fever, arthralgia, and salmon-colored skin rash. Multiorgan involvement is a typical feature. This often causes lots of differential diagnoses and, due to the absence of a definitive diagnostic tool, it is a diagnosis of exclusion. Yamaguchi *et al.* in 1992 and Fautrel *et al*. in 2001 set diagnostic criteria for AOSD which are shown in Table 2 [4, 5].

The skin rash seen in Still’s disease is classically a salmon-pink maculopapular eruption, found most commonly on the upper extremities and trunk, however, face and distal limbs may also be involved. The skin rash may be pruritic. A skin biopsy usually reveals perivascular inflammation of the papillary dermis with lymphohistiocytic inflammation.

Our patient was 22 years of age; he presented with fever of 2 weeks’ duration with arthralgia, myalgia, and generalized lymphadenopathy. As he had fever with arthralgia and myalgia, a viral cause was one of the possibilities. On examination, he had generalized lymphadenopathy with mild hepatosplenomegaly.

His initial workup was performed keeping in mind several differential diagnoses which included systemic bacterial infection, viral hepatitis, scrub typhus, leptospirosis, and even brucellosis. He also had lymphadenopathy and because tuberculosis is quite common in this part of the world, it was also included in the differential diagnosis.

The original criteria to diagnose PUO as proposed by Petersdorf and Beeson included: temperature more than 38.0 °C on multiple occasions for more than 3 weeks and no diagnosis made even after 1 week of hospital admission [11]. The definition has been updated and now the criteria include 3 days of hospital admission for investigations, three OPD visits, or 1 week of intensive ambulatory investigation [12]. This patient had leukocytosis with neutrophilia, with raised ESR and positive CRP. His ADA test was at borderline, a chest X-ray was within normal limits, and a Mantoux test was negative which ruled out tuberculosis. Since there was fever for more than 3 weeks, including 1 week of hospital stay, and routine investigations including cultures were negative, he was evaluated in the line of PUO.

Although he had mildly elevated liver function tests with elevated SGOT and SGPT (154, normal 12–78 IU/L; 210, normal 46–116 IU/L, respectively), serological markers for viral hepatitis were negative. Tests for leptospirosis and brucellosis were also negative along with an HIV test. An ANA test and RA factor were negative which excluded autoimmune disease. Computed tomography of his chest and abdomen were negative for any malignancy. A skin biopsy of our patient was not adequate, and he refused a repeat biopsy. A bone marrow examination was negative for hematological malignancies and *Leishmania donovani* (LD) bodies; granulomas were not seen.

Since, our patient met all the criteria required for the diagnosis of AOSD, a corticosteroid was prescribed. Corticosteroid remains the first-line treatment for AOSD, regardless of the clinical presentation. Usually, prednisolone controls symptoms in approximately 60% of patients with AOSD [4].

After treatment failure with prednisolone and further workup suggesting tuberculosis, treatment with anti-tuberculosis therapy category I was started in our patient. After a few days, he developed skin lesions on his shoulder, back, and face while he was on treatment. This gave the dermatologist the clue for the diagnosis.

ENL is a complication of LL and borderline lepromatous (BL) leprosy. Patients with ENL present with high-grade fever, malaise, and erythematous nodules along with systemic manifestations. Systemic involvement may include arthritis, myositis, lymphadenitis, iritis, neuritis, orchitis, and dactylitis [13]. A patient may also develop anemia, leukocytosis, and have abnormal liver function tests.

Immune complex deposition at various sites is the pathogenesis of ENL along with T cell and macrophage dysregulation with overproduction of tumor necrosis factor [14]. During the era of monotherapy with dapsone, the prevalence of ENL used to be high. With implementation of multidrug therapy for LL and BL the incidence of ENL has decreased [15, 16].

In some individuals, ENL may precede the diagnosis and initiation of therapy. In this case, our patient presented with predominantly systemic signs and symptoms and only developed skin manifestations later [13]. This misleading presentation led to diagnosis of AOSD. Leprosy has been considered AOSD by other authors too. A 17-year-old Chinese boy was referred to a rheumatologist with the diagnosis of AOSD, which upon further investigation was diagnosed as LL with type 2 lepra reaction and treated accordingly [17].

With the diagnosis of AOSD, our patient was prescribed with prednisolone. Steroids inhibit the production of arachidonic acid metabolites, mainly prostaglandins, and further inhibit inflammatory reactions. This led to an improvement in our patient’s clinical signs and symptoms. His fever subsided, and the size of lymph node shrunk. However, when the dose of prednisolone was tapered, the disease again flared up with reactivation of inflammatory process; hence, the reappearance of clinical signs and symptoms.

PUO includes several disease conditions. ENL has been considered a PUO in other studies [18, 19]. During the management of any patient, taking into consideration PUO guides us to think of various etiologies. Since leprosy may present with unusual presentations and sometimes may be a diagnostic dilemma, it should be included as one of the causes of PUO, at least in endemic regions like Nepal.

## Conclusions

Unusual disease presentation can delay its prompt diagnosis and treatment thereby increasing morbidity and mortality. Although Nepal was declared a leprosy-eliminated country in 2010, more than 3000 new cases have been diagnosed annually. This fact reflects that people can still be affected by leprosy and, as a physician, it is important to consider it a differential diagnosis whenever relevant. Furthermore, in an endemic region like Nepal, leprosy should be kept as a possible cause for PUO, which may help to reach the final diagnosis.

## Data Availability

Available.
